# Effects of perspective-taking training based on relational frame theory for cognitive empathy and emotional empathy: Differences in perspective-taking according to various theoretical approaches

**DOI:** 10.1371/journal.pone.0323120

**Published:** 2025-05-09

**Authors:** Yasuhiro Ooshima, Takashi Mitamura

**Affiliations:** 1 Faculty of Human Science, Ritsumeikan University, Osaka, Japan; 2 Department of Psychology, Ritsumeikan University, Osaka, Japan; McGill University Faculty of Arts, CANADA

## Abstract

Perspective-taking has garnered considerable attention across various psychological domains and is often conceptualized as cognitive empathy within the organizational model of empathy. Relational frame theory, a theoretical framework elucidating human behavior and language, addresses perspective-taking and offers corresponding training protocols. However, previous studies have not examined the relationship between training and perspective-taking, defined as cognitive empathy in the organizational model of empathy. This study investigated the impact of perspective-taking training rooted in the relational frame theory on perspective-taking, as conceptualized by the organizational model of empathy. To this end, 43 participants underwent a training regimen and completed pre- and post-training assessments using questionnaires that measure organizational empathy. The post-training scores for the subscales measuring cognitive empathy increased significantly (*p* = 0.031); however, The increase in the score remained significant trend after adjusting for training performance. The scores for some subscales of emotional empathy also increased after training. The limitations of this study include a bias in the sample and discrepancies in the results between scales measuring the same concept. Nonetheless, these results provide scope for debate regarding the impact of perspective-taking training under relational frame theory on perspective-taking within the organizational model of empathy. While considering the limitations of the obtained results, this study discusses the relationship between perspective taking based on relational frame theory and the organizational model of empathy, including emotional empathy.

## Introduction

### Main stream of perspective-taking

Understanding the perspectives of others is important in many social contexts. In psychology, perspective-taking is a key cognitive process that allows an individual to understand the mindsets of others from their own perspective [1]. This phenomenon has garnered considerable scholarly attention, notably within the context of the theory of mind [2] and the three-mountain task [3]. Extensive research has demonstrated the efficacy of interventions involving perspective-taking across diverse domains, ranging from clinical practice to broader social contexts [4,5].

### Perspective-taking and empathy

While perspective-taking has been described as a cognitive product among the concepts involved in understanding others [6,7], the affective dimension has predominantly been explored through empathy. Empathy is defined as feeling the emotions that others evoke as if one were that person or as one’s own emotional response focused on the other [8,9]. Empathy was initially considered an emotional response [10], and as it was differentiated from the cognitive component of understanding others, research has focused exclusively on the emotional aspect [11,12]. Furthermore, practices that predominantly emphasize the emotional aspect of empathy have been developed [8]. Therefore, research on the emotional and cognitive aspects of the perspectives of others has been conducted separately.

### The interactional model for perspective-taking and empathy

The contemporary discourse focuses on the integration and interaction of perspective-taking and empathy. Perspective-taking is formulated as cognitive empathy, and empathy is formulated as emotional empathy [13]. The framework encompassing cognitive empathy (i.e., perspective-taking) and emotional empathy is then formulated as an organizational model of empathy [14]. This framework has become pivotal in contemporary empathy research by elucidating how perspective-taking shapes a broader empathic landscape. For instance, evidence indicates that when empathic concern, an element of emotional empathy, is expressed during perspective-taking, the target of perspective-taking develops favorable feelings toward the perspective-taking party [15]. In other words, simultaneously focusing not only on perspective-taking, which simply involves putting oneself in the other person’s shoes but also on the emotional dimension has a more positive effect on the target of perspective-taking.

### Perspective-taking in relational frame theory

As outlined above, perspective-taking has traditionally been studied with an exclusive focus on its cognitive aspects. Subsequently, research began to incorporate its interaction with emotional dimensions. More recently, perspective-taking has also been approached through relational frame theory (RFT), a linguistic framework, as an extension of its cognitive dimension. RFT understands human language and cognition in context and focuses on these two functional aspects, which are the basis for psychological interventions [16]. RFT elucidates how humans relate words and objects using relational framing, which is grounded in contextual cues rather than in the properties of objects [16]. This approach broadens the scope of understanding and alters the psychological function of stimuli [17]. For example, the word “ball” differs from the actual object, yet the verbal request to “pass me the ball” evokes the behavior of passing the ball, thus influencing human psychology and behavior based on the relationship between words and objects, which are stimuli of distinct natures.

In RFT, perspective-taking refers to the process by which a perspective that distinguishes between the self and others is established through the construction of relational frames [17,18]. This process involves learning three deictic frames that depend on the speaker’s perspective and context and distinguishing between the self and others: I–YOU, HERE–THERE, and NOW–THEN [18]. Learning these three deictic frames fosters the understanding that the self and others are distinct, thereby facilitating the development of the cognitive toreason about others’ perspectives from one’s own [18–20].

Using these deictic frames, RFT has developed a training program aimed at enhancing perspective-taking [19]. This training involves repeated presentation of protocols for reversing the three deictic frames that are based on the speaker’s viewpoint. The reversal process was structured in three levels, according to the relational complexity of the deictic frames: the first level involved a single relation. This protocol contained one of the three frames. Targets were asked to answer questions such as “Yesterday I was watching TV; today I am reading a book. What am I doing now? What was I doing then?” The second level involved a reversed relationship. This protocol contained one of the three frames and was presented once reversed in the form “If I were you and you were me, what would you have?” Targets were asked to answer questions such as “I have a red brick, and you have a green brick. What do you have if I am you and you are me?” The third level involved a double-reversed relationship. This protocol included two of the three frames and presented the two, both reversed, in the form “If now is then, then is now, and here is there, and there is here.” Targets were asked to answer questions such as “Yesterday you sat in the blue chair; now you sit in the black chair. If now is then and then is now, and here is there, and there is here, where are you sitting now? Where would you be sitting then?”

### The gap between RFT and the organizational model of empathy

A questionnaire based on the organizational model of empathy was previously developed [13]. It is capable of measuring emotional empathy and cognitive empathy (i.e., perspective-taking) as separate sub-scales. Accordingly, the questionnaire can comprehensively cover both cognitive and affective aspects of empathy. In contrast, RFT has also developed a training program to enhance perspective-taking [19], which facilitates the cognitive manipulation of switching the positions of oneself and one’s partner using a deictic frame. In other words, while the organizational model of empathy provides a comprehensive framework for understanding both emotional and cognitive empathy [13,14], perspective-taking within RFT focuses primarily on the cognitive aspects (i.e., cognitive empathy). RFT-based training in perspective-taking aims to enhance cognitive skills by facilitating the manipulation of deictic frames, enabling individuals to switch perspectives [4,5], and does not consider the emotional aspect. Just as research based on the organizational model of empathy has primarily focused on communication in adulthood [15], training for perspective-taking within RFT has also been conducted in adulthood in some studies [21]. However, the research has predominantly focused on cognitive development, such as reducing the impact of a fundamental attribution error, without examining the potential interplay with the emotional aspects of empathy. Therefore, while the impact of the interaction between cognitive and emotional empathy, based on the organizational model of empathy, has been empirically examined in communication [15], RFT has only examined the cognitive aspects in the same samples [21]. The relationship between perspective-taking in RFT and models that position perspective-taking as cognitive empathy and assume interaction with emotional empathy has not been examined.

RFT and the organizational model of empathy both contribute to our understanding of perspective-taking but they currently exist as distinct strands of research with limited integration. Bridging this gap by integrating RFT’s perspective-taking with the organizational model of empathy has the potential to enhance our understanding of perspective-taking in RFT by incorporating both cognitive and emotional components. This integration would also expand the application of RFT-based perspective-taking training, making it a more effective intervention for reducing psychological and behavioral issues and improving social communication throughout the lifespan. To achieve these goals, it is important to examine the impact of RFT-based perspective-taking training on both cognitive and emotional empathy and summarize the relationship between RFT and the organizational model of empathy.

### The purpose and the hypothesis of this study

This study examined the effect of perspective-taking training in RFT on perspective-taking (i.e., cognitive empathy) and emotional empathy as formulated in the organizational model of empathy. Specifically, we examined the effects of the training by measuring indices based on the organizational model of empathy before and after training and by assessing changes in the scores. Since perspective-taking training in RFT is oriented toward cognitive operations that promote deictic frameshifting, it was expected to increase only the scores of the subscales related to cognitive aspects in the questionnaire.

## Materials and methods

### Participants

The study included 43 university students (9 men, 34 women, mean age 19.53 ± 1.10 years) enrolled in a private university. Recruitment was organized at the experimenter’s affiliated university, took place online, and utilized a system that incentivizes participation in psychology courses incorporating exercise. The inclusion criteria for this experiment required participants to be 18 or older, following a study by Hooper et al. [21]. To guarantee the accuracy of the experimental results, the exclusion criteria included inadequate access to a stable Internet connection or an environment conducive to concentrating on the experiment, such as a private room. Participant recruitment was conducted from June 15, 2023, to August 1, 2023. Data collection was conducted in parallel, starting on July 1, 2023, and ending on September 1, 2023. The sample size was established as follows. First, we estimated that approximately 40 participants were required, based on Hooper et al. [21]. Next, using the G*Power test power analysis software, we set the risk ratio to 0.05, the power to detect an effect to 0.95, and the effect size to 0.5. We confirmed that approximately 45 participants would be required. A total of 45 people registered to participate in the experiment; of these, two withdrew from the study, and the data collection period ended shortly thereafter; thus, the total number of participants was 43.

### Measures

Face sheet: Participants were asked to provide their gender and age. They were asked to select “male,” “female,” or “other” as their gender.

Interpersonal Reactivity Index Japanese version (IRI-J) [22]: This is a Japanese translation of the Interpersonal Reactivity Index [13]. Comprising 28 items, the IRI-J comprehensively measures empathy across four subscales: personal distress (PD), fantasy (FS), emotional concern (EC), and perspective-taking (PT). PD and EC focus on the emotional aspect of empathy, while PT and FS emphasize the cognitive aspects. Each item is rated on a five-point scale ranging from “1 (not at all true) to “5 (very true).” The scores for each subscale were calculated independently, with higher scores indicating stronger inclinations toward that subscale.

Multidimensional Empathy Scale (MES) [23]: This index was designed to assess the cognitive and emotional dimensions of empathy individually, drawing upon the Interpersonal Reactivity Index (IRI). This scale aims to delineate other- and self-oriented emotional responses. Comprising 24 items, it encompasses five subscales: “Self-orientation,” “Other-orientation,” “Affectedness,” “Perspective-taking,” and “Imagination.” Specifically, “Self-orientation,” “Other-orientation,” and “Affectedness” probe the emotional domain, whereas “Perspective-taking” and “Imagination” explore the cognitive realm. Respondents rated each item on a five-point scale ranging from “not at all true” to “very true.” Higher scores signify a greater inclination within each subscale.

The Three Sense of the Selves Questionnaire (TSSQ) [24]: This index measures “self-as-context,” a concept pertinent to perspective-taking within the RFT framework. It comprises 20 items across four subscales: active and flexible environmental engagement (active), conceptualization, perspective-taking, and awareness of the present moment (present moment). Respondents utilized a seven-point Likert scale, ranging from “1: not at all applicable” to “7: always applicable.” The scores for each subscale were calculated independently, with higher scores indicating a stronger inclination toward that subscale. Higher scores on “conceptualization” indicate a lower level of experience with “self-as-context,” while higher scores for the other three subscales denote a higher level of experience.

### Training

Participants were trained in perspective-taking as formulated in RFT [19]. Specifically, they were repeatedly presented with protocols for reversing the three deictic frames in RFT that depend on the speaker’s viewpoint— “I–YOU,” “HERE–THERE,” and “NOW–THEN”—per the experimenter’s instructions.

Training was conducted according to the procedure proposed by McHugh et al. [19]. With reference to previous studies [19,21], a 36-trial protocol was executed. These trials included six instances of simple relations, 18 instances of reversed relations, and 12 instances of double-reversed relations. These trials were randomly selected, and the presentation order was randomized. Throughout the training, meticulous records were maintained for the response timing and accuracy rates for each protocol. Within this training framework, the occurrence of a single correct response among two questions within one protocol was construed as a chance response [19]. Therefore, only the instances in which both responses were correct were considered accurate. Further details of the protocol are provided in S1 File.

### Procedure

**Fig 1:**
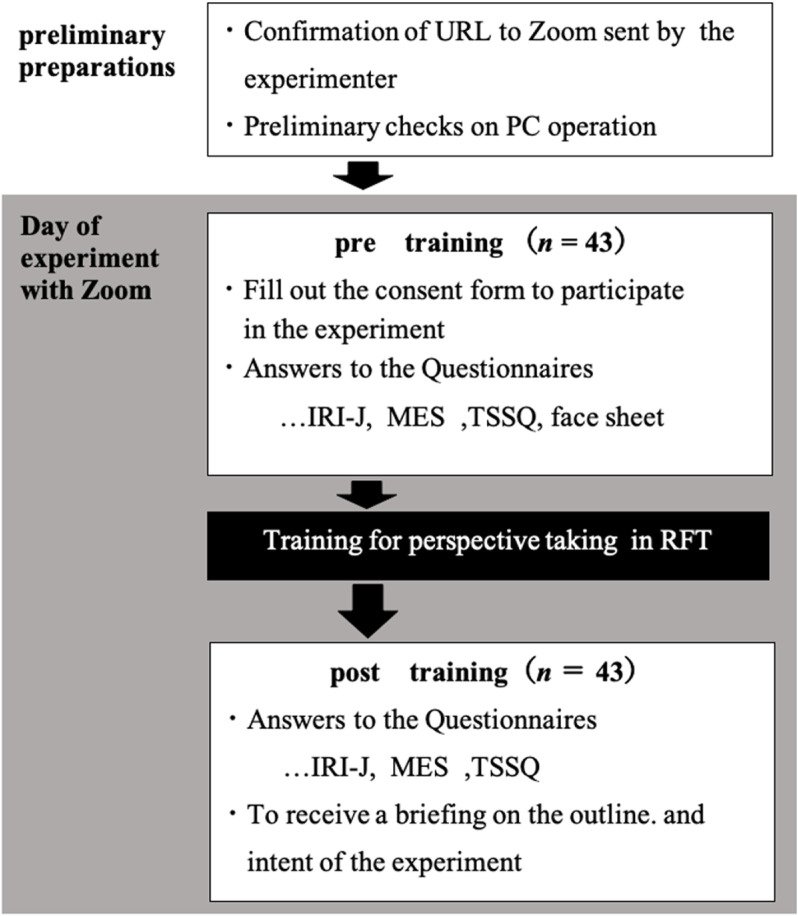
Whole Procedure of the Experiment. IRI-J = Interpersonal Reactivity Index Japanese version. MES = Multidimensional Empathy Scale. TSSQ = The Three Sense of the Selves Questionnaire.

The entire experiment was conducted online using Zoom, a videoconferencing platform (Fig 1). Upon joining Zoom, participants were initially informed of the study’s emphasis on understanding perspectives different from their own. They were also briefed about the training they would receive as part of the study. Subsequently, the experimenter verbally explained the contents of the consent form, and participants provided written consent. The participants completed a questionnaire via the Qualtrics URL, disseminated by the experimenter through the chat function of Zoom, by responding to questions encompassing basic information and the IRI-J, MES, and TSSQ. Following the verification of responses by the experimenter, participants underwent training in perspective-taking.

During training, participants were instructed that the assignment would require them to correctly answer two questions per protocol and informed that they could take as much time as needed to answer the questions. Subsequently, a sample set of questions and answers was provided to facilitate participants’ comprehension of the question framework. Following this, a practice session was conducted, in which the participants responded to five questions. A Q&A session was held after the practice session, as the participants had been informed that inquiries would not be allowed during the main trial but could be addressed thereafter. After confirming that there were no immediate queries, the main trial ensued, comprising 36 randomly presented questions. When two answers were given for a single protocol or participants responded “I don’t know,” the subsequent protocol was initiated. Throughout the interval between the training example and the main trial, both the experimenter and the participants maintained visual contact via the camera. S2 File contains references to the examples and practice sessions.

After undergoing training, the participants completed the Qualtrics questionnaire and IRI-J, MES, and TSSQ. Subsequently, as debriefings, they received explanations regarding the theoretical underpinnings of the training content and its impact. Furthermore, an overview of the overarching purpose of the experiment was provided, including a detailed explanation of the measures administered during the experiment. The experiment and training procedures are stored in protocols.io (dx.doi.org/10.17504/protocols.io.e6nvw1no2lmk/v1).

### Experimental design

Each participant completed the IRI-J, MES, and TSSQ at two-time points: before and after the training sessions. The experiment employed a within-participants design. The number of independent variables was one: the timing of response. This variable had two levels: pre-training (pre) and post-training (post). The dependent variables were the scores for the IRI-J, MES, and TSSQ subscales.

### Analysis

A paired *t*-test was conducted to evaluate the changes in scores for each questionnaire before and after training. For data analysis, the var. test function from the stats package within the R version 4.3.1 was used to assess variance equality across the scales, followed by a *t*-test employing the *t*-test function, both integral components of the package. Cohen’s *d*, the effect size, was calculated by inputting the statistics output by the *t*-test function into R studio. The interpretation of the effect size was set as small, medium, and large effect sizes at approximately 0.2, 0.5, and 0.8, respectively [25]. Training for perspective-taking in RFT was calculated based on the percentage of correct responses and response time. While the thinking process of changing perspectives in training is important, it is possible that aspects of the results, such as the percentage of correct answers and response time, may have an impact on the effects of the training. For example, a case could be made that “the higher the percentage of correct answers, the more effective the training is.” Therefore, we also analyzed the data using a Generalized Linear Mixed Model (GLMM), in which the correct response rate and response time were entered as random effects and the effect of timing was entered as a fixed effect. In each GLMM, pre and post-trainingwere entered as fixed effects, and the scores of each scale were entered as objective variables. There is no agreement on the most appropriate index for measuring the effect size of GLMM [26]. We followed previous studies [27] that defined the effect size and used the marginal *R*^*2*^ (*R*^*2*^*m*), which is the degree to which the data are scattered by only fixed effects, and the conditional *R*^*2*^ (*R*^*2*^*c*), which is the degree to which the data are scattered by both fixed and random effects. In the analysis, GLMM was analyzed using the lmer function from the lmerTest package in the statistical analysis environment R (version 4.3.1), and the effect sizes, *R*^*2*^*m* and *R*^*2*^*c*, were calculated using the r.squaredGLMM function from the MuMIn package. Although there is no consistent standard for the interpretation of the coefficients of determination, including the two coefficients used in this study, we followed the standard commonly used in the field of statistical analysis, which is approximately 0.2, 0.5, and 0.8 for small effect size, medium effect size, and large effect size, respectively. Each code is published in the Open Science Framework.

### Ethical considerations

This research was conducted with the approval of the Ethical Review Committee for Human Subjects Research at Ritsumeikan University. The ethical standards set by this committee were consistent with the American Psychological Association’s (APA) Ethical Principles of Psychologists and Code of Conduct, as well as the Declaration of Helsinki. The ethical code for this study included the following terms: participation was voluntary and could be discontinued if desired; any adverse psychological effects would be treated by a nationally certified psychologist; there would be no disadvantages for not agreeing to participate; and all personal information would be protected, including through data anonymization. Ethical considerations regarding the experiment were reiterated in the consent form and during the verbal explanation at the beginning of the experiment. If the individuals agreed to participate, they signed and submitted the consent forms in front of the experimenter. The signatures were then verified by the experimenter.

## Results

### Data selection

In the data analysis, we initially excluded data from two participants because one participant’s responses were largely incomplete, and measurements for the other participant were incomplete (*n* = 41). For each subcategory of each index, we applied the interquartile range (IQR) method to identify outliers, which are data points that fall outside the IQR. We conducted the data analysis by excluding outliers upon observation. Outliers were excluded if they were observed. An overview of outlier counts revealed four (*n* = 37) for MES Perspective-taking, one (*n* = 40) each for Self-orientation and Other-orientation, and one (*n* = 40) for the conceptualization of the TSSQ. These data are published in the Open Science Framework.

### Results of the paired *t*-tests

Before conducting the paired *t*-tests, we checked for equal variances using the *F*-test (Table 1). Equal variance was not observed missing for Other-orientation, Affectedness, and Imagination subscales of the MES, and Welch’s *t*-tests were applied. For the other subscales, where equal variances were observed, we conducted a normal *t*-test. One-tailed tests were conducted for PT of IRI and Perspective-taking of MES based on the research hypothesis that scores on perspective-taking would increase from pre- to post-training. Table 2 presents the results of paired *t*-tests. Analysis of each IRJ subscale revealed non-significant changes in the mean scores pre- and post-training across all subscales. For EC and FS, Cohen’s *d* effect sizes exceeded 0.2, indicating small effect sizes. Analysis for the MES subscale revealed significant pre- and post-training score changes in Perspective-taking, Other-orientation, and Affectedness. Specifically, perspective-taking exhibited a small effect size, Other-orientation a large effect size, and Affectedness a moderate effect size. No significant change was found in self-orientation and imagination. The TSSQ subscale analysis demonstrated non-significant score changes before and after training across all subscales, with effect sizes below 0.2.

**Table 1:** Results of the F-tests for IRI, MES, TSSQ.

	F-value	CI		df	P-value
		low	high		
IRI-J					
PT	1.00	0.53	1.88	40	0.99
EC	0.93	0.50	1.75	40	0.83
FS	0.83	0.44	1.55	40	0.55
PD	1.05	0.56	1.98	40	0.87
MES					
Perspective Taking	0.82	0.42	1.60	36	0.56
Self-orientation	0.90	0.48	1.70	39	0.74
Other-orientation	0.36	0.19	0.68	39	0.00
Affectedness	0.30	0.16	0.56	40	0.00
Imagination	0.30	0.16	0.56	40	0.00
TSSQ					
Perspective-taking	0.85	0.45	1.59	40	0.60
Conceptualization	1.24	0.65	2.34	39	0.60
Active	0.86	0.46	1.61	40	0.64
Present moment	0.81	0.43	1.52	40	0.51

Note. *SD* = Standard Deviation; IRI-J = Interpersonal Reactivity Index Japanese Edition, PT = Perspective Taking, EC = Empathic Concern, FS = Fantasy, PD = Personal Distress; MES = Multidimensional Empathy Scale; TSSQ = The Three Sense of Selves Questionnaire. *CI* = Confidence Interval. d = Coen’s d. Inf = Infinity.

*df* for all subscales of IRI, Imagination, Affectedness, and subscales of TSSQ except for conceptualization are 42. *df* for Self-orientation, Other-orientation, and conceptualization are 41. *df* for Perspective-taking in MES is 38.

**Table 2:** Results of the Paired *t* -test for IRI, MES, TSSQ.

	Pre-Training		Post-training		T-value (95% CI)	P-value	Effect size (d)
	mean	SD	mean	SD			
IRI-J							
PT	26.10	4.39	25.73	4.38	-0.77 (-1.16: Inf)	0.779	0.12
EC	21.90	3.56	22.34	3.69	1.39 (-0.20: 1.08)	0.173	0.22
FS	24.88	6.01	24.07	6.61	-1.64 (-1.80: 0.19)	0.110	0.26
PD	23.80	5.94	24.12	5.79	0.54 (-0.87: 1.51)	0.593	0.08
MES							
Perspective-taking	18.43	2.05	19.05	2.26	1.93 (0.08: Inf)	0.031	0.33
Self-orientation	15.58	2.48	15.33	2.62	-1.11 (-0.71: 0.21)	0.275	0.18
Other-orientation	16.88	1.71	19.68	2.85	5.98 (1.85: 3.75)	0.000	0.94
Imagination	18.54	2.49	18.27	4.57	`-0.58 (-1.21: 0.67)	0.567	0.09
Affectedness	15.93	2.51	18.10	4.60	` 3.70 (0.98: 3.36)	0.001	0.58
TSSQ							
Perspective-taking	13.73	3.69	13.39	4.02	`-0.97 (-1.05: 0.37)	0.336	0.15
Conceptualization	29.93	4.58	30.10	4.11	` 0.51 (-0.52: 0.87)	0.613	0.08
Active	33.71	6.62	33.12	7.13	`-1.23 (-1.55: 0.38)	0.226	0.19
Present moment	16.37	4.43	16.34	4.92	`-0.08 (-0.63: 0.58)	0.936	0.01

Note. *SD* = Standard Deviation; IRI-J = Interpersonal Reactivity Index Japanese Edition, PT = Perspective Taking, EC = Empathic Concern, FS = Fantasy, PD = Personal Distress; MES = Multidimensional Empathy Scale; TSSQ = The Three Sense of Selves Questionnaire. *CI* = Confidence Interval. d = Coen’s d. Inf = Infinity.

*df* for all subscales of IRI, Imagination, Affectedness, and subscales of TSSQ except for conceptualization are 42. *df* for Self-orientation, Other-orientation, and conceptualization are 41. *df* for Perspective-taking in MES is 38.

### GLMM results

Tables 3 and 4 display the outcomes of each GLMM in which the correct response rate and response time were entered separately as random effects, whereas the response timing was considered a fixed effect. In the GLMM, with the correct response rate as a random effect, significant fixed effects were noted for the Other-orientation and Affectedness of the MES. For effect size, the *R*^*2*^*c* values were moderate for Other-orientation only. In the GLMM, with response time as a random effect, there was a significant tendency for perspective-taking in MES. Furthermore, the fixed effects were significant for the Other-orientation and Affectedness. Regarding effect sizes, the *R*^*2*^*c* for Other-orientation and Affectedness were moderate at around 0.5.

**Table 3:** Results of GLMM with Accuracy Rate as a Random Effect.

	β	95% CI	SE	T-value	P-value	Effect Size	
						R2m	R2c
IRI-J							
PT	−0.37	(−2.22: 1.48)	0.94	−0.39	0.698	0.00	0.06
EC	0.44	(−0.92: 1.80)	0.69	0.64	0.525	0.00	0.31
FS	−0.80	(−3.17: 1.56)	1.19	−0.67	0.503	0.00	0.33
PD	0.32	(−2.10: 2.74)	1.23	0.26	0.797	0.00	0.11
MES							
Perspective-taking	0.62	(−0.21: 1.45)	0.42	1.49	0.144	0.02	0.40
Self-orientation	−0.25	(−1.28: 0.78)	0.52	−0.48	0.633	0.00	0.19
Other-orientation	2.80	(1.87: 3.73)	0.47	5.97	0.000	0.24	0.46
Imagination	−0.27	(−1.62: 1.08)	0.68	−0.39	0.695	0.00	0.36
Affectedness	2.17	(0.57: 3.78)	0.80	2.71	0.009	0.08	0.13
TSSQ							
Perspective-taking	−0.34	(−2.01: 1.33)	0.85	−0.40	0.690	0.00	0.01
Conceptualization	0.18	(−1.66: 2.01)	0.92	0.19	0.851	0.00	0.12
Active	−0.59	(−3.46: 2.29)	1.44	−0.41	0.686	0.00	0.12
Present moment	−0.02	(−1.58: 1.53)	0.79	−0.03	0.975	0.00	0.49

Note. *SD* = Standard Deviation; IRI-J = Interpersonal Reactivity Index Japanese Edition, PT = Perspective Taking, EC = Empathic Concern, FS = Fantasy, PD = Personal Distress; MES = Multidimensional Empathy Scale; TSSQ = The Three Sense of Selves Questionnaire. *CI* = Confidence Interval; *SE* = Standard Error.

*R2m* means the variance explained by fixed effect. *R2c* means the variance explained by fixed effect and random effect.

*n* = 41 for all subscales of IRI, Imagination, Affectedness, and subscales of TSSQ except for conceptualization. *n* = 40 for Self-orientation, Other-orientation and conceptualization; *n* = 37 for Perspective-taking in MES.

**Table 4:** Results of GLMM with Response Time as a Random Effect.

	β	95% CI	SE	T-value	P-value	Effect Size	
						R2m	R2c
IRI-J							
PT	−0.37	(−1.30: 0.57)	0.47	−0.78	0.443	0.00	0.76
EC	0.44	(−0.188: 1.07)	0.32	1.39	0.173	0.00	0.84
FS	−0.80	(−1.78: 0.17)	0.49	−1.64	0.109	0.00	0.88
PD	0.32	(−0.85: 1.48)	0.59	0.54	0.593	0.00	0.79
MES							
Perspective-taking	0.62	(−0.02: 1.26)	0.32	1.93	0.062	0.02	0.59
Self-orientation	−0.25	(−0.70: 0.20)	0.23	−1.11	0.275	0.00	0.84
Other-orientation	2.80	(1.87: 3.73)	0.47	5.98	0.000	0.26	0.42
Imagination	−0.27	(−1.19: 0.65)	0.46	−0.58	0.567	0.00	0.67
Affectedness	2.17	(1.01: 3.33)	0.59	3.70	0.001	0.08	0.53
TSSQ							
Perspective-taking	−0.34	(−1.04: 0.35)	0.35	−0.98	0.336	0.00	0.83
Conceptualization	0.18	(−0.51: 0.86)	0.34	0.51	0.613	0.00	0.88
Active	−0.59	(−1.53: 0.36)	0.48	−1.23	0.226	0.00	0.90
Present moment	−0.02	(−0.62: 0.57)	0.30	−0.08	0.936	0.00	0.91

Note. *SD* = Standard Deviation; IRI-J = Interpersonal Reactivity Index Japanese Edition, PT = Perspective Taking, EC = Empathic Concern, FS = Fantasy, PD = Personal Distress; MES = Multidimensional Empathy Scale; TSSQ = The Three Sense of Selves Questionnaire. *CI* = Confidence Interval; *SE* = Standard Error.

*R2m* means the variance explained by fixed effect. *R2c* means the variance explained by fixed effect and random effect.

*n* = 41 for all subscales of IRI, Imagination, Affectedness and subscales of TSSQ except for conceptualization. *n* = 40 for Self-orientation, Other-orientation and conceptualization; *n* = 37 for Perspective-taking in MES.

## Discussion

The present study examined the effect of perspective-taking training in RFT on perspective-taking (i.e., cognitive empathy) and emotional empathy as formulated in the organizational model of empathy. Training for perspective-taking in RFT involved the cognitive manipulation of shifting the perspective through promoting deictic framing. Therefore, it is classified as cognitive empathy in the organizational model of empathy; hence, it was predicted that the effect of the training would affect only cognitive empathy in the organizational model of empathy.

### Interpretations of training effects for cognitive empathy

The paired t-test results showed that MES Perspective-taking scores increased significantly before and after the training. The effect size was small. In GLMM, perspective-taking scores only showed a significant trend in the pre-and post-training periods when response time was entered as a random effect, and its variability was considered. These results partially support the hypothesis regarding the impact of RFT training on cognitive empathy (i.e., perspective-taking) within the organizational model of empathy. They are consistent with findings from prior studies by Montoya-Rodríguez et al. and Hooper et al. [4,5,21]. However, after controlling for various variables, the effects showed significant trends, raising questions regarding the interpretation of the results.

One of the reasons the effect of the training on perspective-taking in RFT on MES Perspective-taking was not significant when considering response time and the correct response rate is that there were nuanced differences between the nature of the training and the content of the MES Perspective-taking items. A characteristic feature of the MES Perspective-taking items is that they assume a real-life situation in which the individual is actually communicating with others [23]. However, in training for perspective-taking in RFT, although it is desirable to imagine the scene as real, only an imaginary scene is assumed. In fact, for perspective-taking exercises based on RFT, imagining trusted others in everyday life has also been shown to be more effective in skills such as reducing emotional discomfort [28]. Therefore, the difference in whether the training is assumed to be an imaginary or realistic scene is directly related to the ease of connection with Perspective-taking in the MES. In addition, it is thought that participants who responded to the training assuming that they were in a real-life situation were more likely to respond accurately and quickly due to the ease of visualizing the scene and that this would have a positive spillover effect on the MES Perspective-taking scores. In brief, the degree to which participants trained under the assumption of a real situation was reflected in the response time and rate of correct responses, and only those participants who performed well in these two areas improved MES Perspective-taking scores.

### Interpretations of training effects for other-orientation

Although not initially anticipated, Other-orientation scores, which partly measure emotional empathy, showed significant increases both in the paired t-test and the GLMM analysis. The paired t-test indicated a large effect size for the increase in scores from before to after training, suggesting a substantial improvement in participants’ ability to consider the perspectives of others. The GLMM results further supported this finding, demonstrating that the change in Other-orientation scores was significantly predicted in the pre-and post-training periods with a medium effect size. This indicates that the training had a considerable and lasting impact on participants’ other orientations, even when accounting for variations in response rates and response time. The relationship between perspective-taking in RFT and the affective aspects of understanding others has not been previously examined, and it would be worthwhile to discuss this relationship in light of the above results.

As RFT training did not affect Self-orientation but affected Other-orientation, it is likely associated with other-oriented emotion. Although no empirical studies have focused on RFT and empathy, RFT has been reported to contribute to conceptualizing the current situation in which empathic emotions emerge among individuals [29]. In this process, the distinction and shift of self and others through deictic frames such as “I-YOU” is emphasized [19]. Because of those shifting perspectives, the other-oriented emotion may be strengthened while incorporating the other’s perspectives. In addition, a positive correlation between other-oriented emotion and social skills has been shown [30], and RFT training also aims to improve such social skills [4,5]. Based on the above, it is considered that both RFT and empathy are related in the sense that they are skills and that the process of shifting perspectives between the self and others during the training contributed to the increase in other-oriented thinking.

### Interpretations of training effects for affectedness

Similar to Other-orientation, Affectedness scores, which also partly measure emotional empathy, showed significant increases in both the paired t-test and GLMM analysis. The paired t-test revealed a moderate effect size for the increase in scores, suggesting a noticeable improvement in participants’ emotional responsiveness. The GLMM results confirmed this finding, indicating that the change in Affectedness scores was significantly predicted in the pre-and post-training periods with a moderate effect size. Notably, the entire model was explained by considering the variability in response time, suggesting that changes in response time might be a key factor in understanding the impact of the training on Affectedness.

The increase in Affectedness scores related to emotional empathy after perspective-taking training in RFT can be attributed to the fact that sensitivity to others temporarily increased due to rapid exposure to opportunities to imagine the position of others because of having been trained to switch between the self and others. Affectedness refers to the ease of being involved in the psychological state of others [23]. When this tendency is high, sensitivity to the position and psychological state of others increases, meaning that people are more likely to be affected. Training for perspective-taking in RFT switches the perspectives of self and others [19] through the process of adopting the perspective of others via the transformation of stimulus functions, transforming what is happening to others as if it is happening to the self [20]. Moreover, it has been suggested that, through switching perspectives between oneself and others, it is easier to feel the emotions of others, such as pain, as if they were one’s own emotions [31]. Thus, perspective-taking training should include a process that crosses the boundary between the self and others, and this process is thought to increase sensitivity to others.

### Overview of the training effects for emotional empathy

However, based on the results of this study, in which Self-orientation did not change but Other-orientation and Affectedness did, we cannot conclude that RFT perspective-taking training affects overall emotional empathy. Previous research suggested that empathy is created through the mechanism of looking at the emotions of others through the emotions of the self and that the involvement of Self- and Other-orientation is necessary for emotional empathy [32,33]. Since Affectedness is also derived from personal distress [34], which prioritizes the reduction of one’s psychological distress [35,36] and inhibits empathic communication [14], Affectedness alone does not positively affect emotional empathy. Therefore, comprehensive changes in Self-orientation, Other-orientation, and Affectedness are required to conclude that perspective-taking training in RFT affects emotional empathy.

### Limitations

This study has three primary limitations. First, the IRI-J failed to capture the impact of perspective-taking training on RFT across all subscales, leaving the interpretation of the MES results subject to ongoing debate. The IRI-J, a Japanese adaptation of the original IRI [13] led to the development of the MES to rectify the Japanese version. Consequently, the MES and IRI-J exhibited significant similarities as scales, necessitating careful consideration when interpreting the MES results. Second, no effect of training was observed on the TSSQ, which measures “self-as-context,” a concept related to perspective-taking as formulated in RFT. Although a change in the scores in this study was expected, no change was observed. Therefore, it was necessary to reconsider the relationship between training and the “self-as-context”; however, this was not the focus of the present study. Finally, the study sample exhibited significant bias. This experiment primarily comprised female participants, potentially introducing unforeseen confounding variables related to sex differences. Additionally, the sample was limited to university students, limiting the generalizability of the findings to broader populations. Using a more diverse population in future experiments will enhance the validity of the results, particularly after adjusting for these variables.

## Conclusions

The results of this study provide important suggestions for developing future perspective-taking training and reconsidering program content. It is possible that the effect of RFT training on perspective-taking in the MES was moderated by various factors, such as differences in participants’ ability to connect everyday situations and experimental situations. Therefore, it is necessary to modify the content of the training program to ensure that it is relevant to the trainees’ daily lives and increase the concreteness of the images during training for the purpose of wider clinical application. In addition, this study found a link between perspective-taking in RFT with a strong cognitive orientation and perspective-taking formulated in an organizational model of empathy that encompasses emotional processes, which contribute to the organization of the perspective-taking concept in psychology. However, we found that perspective-taking training in RFT also affected emotional aspects, such as Other-orientation and Affectedness. The relationship between perspective-taking and these affective aspects requires further investigation. Therefore, future research should consider the following.

First, the scenario used in the training developed in McHugh et al. [19] should be adapted from fictional situations to situations that are more relevant to participants’ daily lives. Next, the effect of the training on scales that measure cognitive empathy and emotional empathy should be confirmed, similar to the approach adopted in this study. Furthermore, in the training developed by McHugh et al. [19], the sensitivity to the emotional aspects of others increased through the process of switching between one’s own and others’ perspectives. Therefore, it is necessary to measure the degree of sensitivity to emotional aspects and to confirm whether it mediates the change in emotional empathy. Additionally, the findings from these two perspectives should be integrated to develop novel training methods for perspective taking. Finally, the effectiveness of these new methods should be verified.

## Supporting information

S1 File Appendix A. The file lists 36 training protocols for perspective-taking in RFT conducted during the actual trial, presented in order.
(DOCX)

S2 File Appendix B. The file contains one p protocol as an example and three protocols for practice trials. https://journals.plos.org/plosone/s/submit-now.
(DOCX)
