# Supplementary material for: Effects of perspective-taking training based on relational frame theory for cognitive empathy and emotional empathy: Differences in perspective-taking according to various theoretical approaches
Source: PLoS One. 2025 May 9;20(5):e0323120. doi: 10.1371/journal.pone.0323120 (PMC12063820; doi:10.1371/journal.pone.0323120)
Supplement: S1 File — The file lists 36 training protocols for perspective-taking in RFT conducted during the actual trial, presented in order. (DOCX) [file pone.0323120.s001.docx]

**Appendix A**

The original version is attached at the bottom of each japanese protocol.

1. 私は昨日は読書をしており、今日はテレビを見ています。その時は、私は何をしていましたか?私は今何をしているのでしょうか?

Yesterday I was watching television, today I am reading. What was I doing then? What am I doing now?

1. 私はここにある青い椅子に座り、あなたはそこにある黒い椅子に座っています。もし、ここがそこであり、そこがここであったとしたら。あなたはどこに座っていますか?私はどこに座っていますか?

I am sitting here on the blue chair and you are sitting there on the black chair. If here was there and there was here. Where would YOU be sitting? Where would I be sitting?

1. 私はここにある黒い椅子に座り、あなたはそこにある青い椅子に座っています。もし私があなたであなたが私であったとしたら、そしてここがそこであそこがここだったとしたら。私はどこに座っていますか?あなたはどこに座っていますか?

I am sitting here on the black chair and you are sitting there on the blue chair. If I was you and you were me and if here was there and there was here. Where would I be sitting? Where would YOU be sitting?

1. あなたは昨日そこにある青い椅子に座り、あなたは今日ここにある黒い椅子に座っています。もし、ここがそこでありそこがここであったとしたら、そして今がそのときでありそのときが今であったとしたら。あなたはそのときどこに座っていましたか?あなたは今どこに座っていますか?

Yesterday you were sitting there on the blue chair, today you are sitting here on the black chair. If here was there and there was here and if now was then and then was now. Where would you be sitting then? Where would you be sitting now?

1. 私は昨日そこにある黒い椅子に座り、私は今日ここにある青い椅子に座っています。もし、ここがそこでありそこがここであったとしたら、そして今がそのときでありそのときが今であったとしたら。私はそのときどこに座っていましたか?私は今どこに座っていますか?

Yesterday I was sitting there on the black chair, today I am sitting here on the blue chair. If here was there and there was here and if now was then and then was now. Where would I be sitting then? Where would I be sitting now?

1. 私はここにある青い椅子に座り、あなたはそこにある黒い椅子に座っています。もし私があなたで、あなたが私だったら。あなたはどこに座っていますか?私はどこに座っていますか?

I am sitting here on the blue chair and you are sitting there on the black chair. If I was you and you were me. Where would YOU be sitting? Where would I be sitting?

1. 私はここにある黒い椅子に座り、あなたはそこにある青い椅子に座っています。もし私があなたで、あなたが私だったら。あなたはどこに座っていますか?私はどこに座っていますか?

I am sitting here on the black chair and you are sitting there on the blue chair. If I was you and you were me. Where would YOU be sitting? Where would I be sitting?

1. あなたは昨日本を読んでいて、あなたは今日テレビを見ています。もし今がそのときで、そのときが今であったとしたら。あなたは今何をしていますか?その時、あなたは何をしていましたか?

Yesterday you were reading, today you are watching television. If now was then and then was now. What would you be doing now? What were you doing then?

1. 私は昨日、そこにある黒い椅子に座り、今日はここにある青い椅子に座っています。もしここがそこであり、そこがここであったとしたら。私は今どこに座っていますか?私はそのときどこに座っていましたか?

I was sitting there on the black chair and I am sitting here on the blue chair. If here was there and there was here. Where would I be sitting now? Where would I be sitting then?

1. 私はここにある青い椅子に座り、あなたはそこにある黒い椅子に座っています。もし私があなたであなたが私であったとしたら、そしてここがそこであそこがここだったとしたら。あなたはどこに座っていますか?私はどこに座っていますか?

I am sitting here on the blue chair and you are sitting there on the black chair. If I was you and you were me and if here was there and there was here.Where would YOU be sitting? Where would I be sitting?

1. 私はここにある黒い椅子に座り、あなたはそこにある青い椅子に座っています。あなたはどこに座っていますか?私はどこに座っていますか?

I am sitting here on the black chair and you are sitting there on the blue chair. Where are YOU sitting? Where am I sitting?

1. 私はここにある青い椅子に座り、あなたはそこにある黒い椅子に座っています。もし私があなたであなたが私であったとしたら、そしてここがそこであそこがここだったとしたら。私はどこに座っていますか?あなたはどこに座っていますか?

I am sitting here on the blue chair and you are sitting there on the black chair. If I was you and you were me and if here was there and there was here. Where would I be sitting? Where would YOU be sitting?

1. 私は昨日はテレビを見ており、今日は読書をしています。私は今何をしているのでしょうか?その時は、私は何をしていましたか?

Yesterday I was watching television, today I am reading. What am I doing now? What was I doing then?

1. 私はここにある青い椅子に座り、あなたはそこにある黒い椅子に座っています。もし私があなたで、あなたが私だったら。私はどこに座っていますか?あなたはどこに座っていますか?

I am sitting here on the blue chair and you are sitting there on the black chair. If I was you and you were me. Where would I be sitting? Where would YOU be sitting?

1. あなたは昨日、そこにある青い椅子に座り、あなたは今日ここにある黒い椅子に座っています。もし、ここがそこであり、そこがここであったとしたら。そのときあなたはどこに座っていましたか?あなたは今どこに座っていますか?

Yesterday you were sitting there on the blue chair, today you are sitting here on the black chair. If here was there and there was here. Where were you sitting then? Where would you be sitting now?

1. あなたは昨日、そこにある青い椅子に座り、あなたは今日ここにある黒い椅子に座っています。もし、ここがそこであり、そこがここであったとしたら。あなたは今どこに座っていますか?あなたはそのときどこに座っていましたか?

Yesterday you were sitting there on the blue chair, today you are sitting here on the black chair. If here was there and there was here. Where would you be sitting now? Where were you sitting then?

1. あなたは昨日そこにある青い椅子に座り、あなたは今日ここにある黒い椅子に座っています。もし、ここがそこでありそこがここであったとしたら、そして今がそのときでありそのときが今であったとしたら。あなたは今どこに座っていますか?あなたはそのときどこに座っていましたか?

Yesterday you were sitting there on the blue chair, today you are sitting here on the black chair. If here was there and there was here and if now was then and then was now. Where would you be sitting then? Where would you be sitting now?

1. 私は昨日本を読んでいて、私は今日テレビを見ています。もし今がそのときで、そのときが今であったとしたら。私は今何をしていますか?私はそのとき何をしていましたか?

Yesterday I was reading, today I am watching television. If now was then and then was now. What would I be doing now?What was I doing then?

1. あなたは昨日そこにある黒い椅子に座り、あなたは今日ここにある青い椅子に座っています。もし、ここがそこでありそこがここであったとしたら、そして今がそのときでありそのときが今であったとしたら。あなたは今どこに座っていますか?あなたはそのときどこに座っていましたか?

Yesterday you were sitting there on the black chair, today you are sitting here on the blue chair. If here was there and there was here and if now was then and then was now. Where would you be sitting now? Where would you be sitting then?

1. 私は昨日本を読んでいて、私は今日テレビを見ています。もし今がそのときで、そのときが今であったとしたら。私はそのとき何をしていましたか?今、私は何をしていますか?

Yesterday I was reading, today I am watching television. If now was then and then was now. What was I doing then? What would I be doing now?

1. 私は赤いレンガを持っていて、あなたは緑のレンガを持っています。私はどちらのレンガを持っていますか?あなたはどちらのレンガを持ってますか?

I have a red brick and you have a green brick. Which brick do I have?Which brick do YOU have?

1. 私は昨日そこにある黒い椅子に座り、私は今日ここにある青い椅子に座っています。もし、ここがそこでありそこがここであったとしたら、そして今がそのときでありそのときが今であったとしたら。私は今どこに座っていますか?私はそのときどこに座っていましたか?

Yesterday I was sitting there on the black chair, today I am sitting here on the blue chair. If here was there and there was here and if now was then and then was now.

Where would I be sitting now? Where would I be sitting then?

1. 私は昨日そこにある青い椅子に座り、私は今日ここにある黒い椅子に座っています。もし、ここがそこでありそこがここであったとしたら、そして今がそのときでありそのときが今であったとしたら。私はそのときどこに座っていましたか?私は今どこに座っていますか?

Yesterday I was sitting there on the blue chair, today I am sitting here on the black chair. If here was there and there was here and if now was then and then was now. Where would I be sitting then? Where would I be sitting now?

1. あなたは昨日そこにある黒い椅子に座り、あなたは今日ここにある青い椅子に座っています。もし、ここがそこでありそこがここであったとしたら、そして今がそのときでありそのときが今であったとしたら。あなたはそのときどこに座っていましたか?あなたは今どこに座っていますか?

Yesterday you were sitting there on the black chair, today you are sitting here on the blue chair. If here was there and there was here and if now was then and then was now. Where would you be sitting then? Where would you be sitting now?

1. 私はここにある黒い椅子に座り、あなたはそこにある青い椅子に座っています。もし、ここがそこであり、そこがここであったとしたら。あなたはどこに座っていますか?私はどこに座っていますか?

I am sitting here on the black chair and you are sitting there on the blue chair. If I was you and you were me. Where would YOU be sitting? Where would I be sitting?

1. 私はここにある黒い椅子に座り、あなたはそこにある青い椅子に座っています。もし、ここがそこであり、そこがここであったとしたら。私はどこに座っていますか?あなたはどこに座っていますか?

I am sitting here on the black chair and you are sitting there on the blue chair. If I was you and you were me. Where would I be sitting? Where would YOU be sitting?

1. 私は昨日、そこにある青い椅子に座り、今日はここにある黒い椅子に座っています。もしここがそこであり、そこがここであったとしたら。私は今どこに座っていますか?私はそのときどこに座っていましたか?

Yesterday I was sitting there on the blue chair, today I am sitting here on the black chair. If here was there and there was here. Where would I be sitting now? Where was I sitting then?

1. あなたは昨日、そこにある黒い椅子に座り、あなたは今日ここにある青い椅子に座っています。もし、ここがそこであり、そこがここであったとしたら。あなたは今どこに座っていますか?あなたはそのときどこに座っていましたか?

Yesterday you were sitting there on the black chair, today you are sitting here on the blue chair. If here was there and there was here. Where would you be sitting now? Where were you sitting then?

1. 私は昨日そこにある青い椅子に座り、私は今日ここにある黒い椅子に座っています。もし、ここがそこでありそこがここであったとしたら、そして今がそのときでありそのときが今であったとしたら。私は今どこに座っていますか?私はそのときどこに座っていましたか?

Yesterday I was sitting there on the blue chair, today I am sitlting here on the black chair. If here was there and there was here and if now was then and then was now. Where would I be sitting then? Where would I be sitting now?

1. 私はここにある黒い椅子に座り、あなたはそこにある青い椅子に座っています。もし私があなたであなたが私であったとしたら、そしてここがそこであそこがここだったとしたら。あなたはどこに座っていますか?私はどこに座っていますか?

I am sitting here on the black chair and you are sitting there on the blue chair. If I was you and you were me and if here was there and there was here. Where would YOU be sitting? Where would I be sitting?

1. あなたは昨日テレビを見ていて、あなた今日本を読んでいます。もし今がそのときで、そのときが今であったとしたら。あなたは今何をしていますか?あなたはそのとき何をしていましたか?

Yesterday you were watching television, today you are reading. If now was then and then was now. What would you be doing now? What were you doing then?

1. 私は緑色のレンガを持っていて、あなたは赤色のレンガを持っています。あなたはどちらのレンガを持っていますか?私はどちらのレンガを持っているのでしょうか?

I have a green brick and you have a red brick. Which brick do YOU have? Which brick do I have?

1. あなたは昨日そこにある黒い椅子に座り、あなたは今日ここにある青い椅子に座っています。もし今がそのときで、そのときが今であったとしたら。あなたは今どこに座っていますか?そのときあなたはどこに座っていましたか?

Yesterday you were sitting there on the black chair, today you are sitting here on the blue chair. If now was then and then was now. Where would you be sitting now? Where were you sitting then?

1. 私は昨日テレビを見ていて、私は今日本を読んでいます。もし今がそのときで、そのときが今であったとしたら。私は今何をしていますか?私はそのとき何をしていましたか?

Yesterday I was watching television, today I am reading. If now was then and then was now. What was I doing now? What would I be doing then?

1. 私はここにある青い椅子に座り、あなたはそこにある黒い椅子に座っています。私はどこに座っていますか?あなたはどこに座っていますか?

I am sitting here on the blue chair and you are sitting there on the black chair. Where am I sitting? Where are YOU sitting?

1. 私は昨日、そこにある青い椅子に座り、今日はここにある黒い椅子に座っています。もしここがそこであり、そこがここであったとしたら。そのとき私はどこに座っていましたか?私は今どこに座っていますか?

Yesterday I was sitting there on the blue chair, today I am sitting here on the black chair. If here was there and there was here. Where was I sitting then? Where would I be sitting now?
