# Supplementary material for: Effects of perspective-taking training based on relational frame theory for cognitive empathy and emotional empathy: Differences in perspective-taking according to various theoretical approaches
Source: PLoS One. 2025 May 9;20(5):e0323120. doi: 10.1371/journal.pone.0323120 (PMC12063820; doi:10.1371/journal.pone.0323120)
Supplement: S2 File — The file contains one p protocol as an example and three protocols for practice trials. https://journals.plos.org/plosone/s/submit-now. (DOCX) [file pone.0323120.s002.docx]

**Appendix B**

The original version is attached at the bottom of each japanese protocol.

**The protocols presented as the example**

私は昨日はテレビを見ており、今日は読書をしています。私は今何をしていますか？そのとき、私は何をしていましたか？

Yesterday I was watching television, today I am reading.

What am I doing now?

What was I doing then?

**The protocols presented in the practice session**

私は緑色のレンガ、あなたは赤色のレンガを持っています。もし私があなたで、あなたが私だったら、どのレンガをあなたは持っていますか？私はどのレンガを持っていいますか？

I have a green brick and you have a red brick. If I was you and you were me.

Which brick would YOU have?

Which brick would I have?

昨日、あなたはそこの青い椅子に座り、今日、あなたはここの黒い椅子に座っています。もし今がそのときで、そのときが今だとしたら。あなたは今どこに座っていますか？そのとき、あなたはどこに座っていましたか？

Yesterday you were sitting there on the blue chair, today you are sitting here on the black chair. If now was then and then was now.

Where would you be sitting now?

Where were you sitting then?

昨日、あなたはそこの黒い椅子に座り、今日、あなたはここの青い椅子に座っています。もし今がそのときで、そのときが今だとしたら。そのときあなたはどこに座っていましたか？今、あなたはどこに座っていますか？

Yesterday you were sitting there on the black chair, today you are sitting here on the blue chair. If now was then and then was now.

Where were you sitting then?

Where would you be sitting now?
